# A Biomechanical Analysis of Muscle Force Changes After Bilateral Sagittal Split Osteotomy

**DOI:** 10.3389/fphys.2021.679644

**Published:** 2021-06-03

**Authors:** Dominik Pachnicz, Przemysław Stróżyk

**Affiliations:** ^1^Faculty of Mechanical Engineering, Wrocław University of Science and Technology, Wrocław, Poland; ^2^Department of Mechanics, Materials and Biomedical Engineering, Wrocław University of Science and Technology, Wrocław, Poland

**Keywords:** mandible, computer simulation, elevator muscles, sagittal split ramus osteotomy, muscle tension

## Abstract

A basic procedure affecting maxillofacial geometry is the bilateral sagittal split osteotomy. During the surgery, the bony segments are placed in a new position that provides the correct occlusion. Changes in the geometry of the mandible will affect the surrounding structures and will have a significant impact on the functioning of the masticatory system. As a result of the displacement of the bone segment, the biomechanical conditions change, i.e., the load and the position of the muscles. The primary aim of this study was to determine the changes in the values of the muscular forces caused by mandible geometry alteration. The study considered the translation and rotation of the distal segment, as well as rotations of the proximal segments in three axes. Calculations were performed for the unilateral, static loading of a model based on rigid body mechanics. Muscles were modeled as spring elements, and a novel approach was used to determine muscle stiffness. In addition, an attempt was made, based on the results obtained for single displacements separately, to determine the changes in muscle forces for geometries with complex displacements. Based on the analysis of the results, it was shown that changes in the geometry of the mandibular bone associated with the bilateral sagittal split osteotomy will have a significant effect on the values of the masticatory muscle forces. Displacement of the distal segment has the greatest effect from −21.69 to 26.11%, while the proximal segment rotations affected muscle force values to a less extent, rarely exceeding 1%. For Yaw and Pitch rotations, the opposite effect of changes within one muscle is noticed. Changes in muscle forces for complex geometry changes can be determined with a high degree of accuracy by the appropriate summation of results obtained for simple cases.

## Introduction

Changes in the geometry of the craniofacial bones affect the surrounding structures and have a significant impact on the functioning of the masticatory system (Grunheid et al., 2009). Moreover, a relationship between the shape of the lower jaw and muscle forces has been emphasized in several works (Van Spronsen et al., 1997; Sella-Tunis et al., 2018).

The identification of muscle forces, as well as temporomandibular joint (TMJ) loading, has been the subject of many analyses (Pruim et al., 1980; Throckmorton and Throckmorton, 1985; Koolstra and van Eijden, 1992; Osborn, 1995; Peck et al., 2000; Iwasaki et al., 2004; Sellers and Crompton, 2004; Schindler et al., 2007; Hannam et al., 2008; Kijak et al., 2015). No direct force measurement method has yet been developed. The available considerations are based on the calculations and measurements of parameters that are indirectly related to the capability of muscles to produce force. The main methods used to measure muscle activity include electromyography (EMG) (Van Eijden, 1990; Iwasaki et al., 2004), and the maximum magnitude of muscle contraction force or maximum muscular capacity (MMC) can be determined from CT measurements (O’Connor et al., 2005). The cross-sectional area is usually multiplied by the constant intrinsic strength of skeletal muscle λ, which can range from 9 N/cm^2^ to even 140 N/cm^2^, depending on the publication. In the vast majority of works, however, it is in the range of 30–40 N/cm^2^ (Pruim et al., 1980; Zajac, 1989; Peck et al., 2000; Hattori et al., 2003; Zheng et al., 2019). The obtained values after appropriate scaling are often used in analyses, comparisons, or even numerical simulations that reproduce the loading conditions of the stomatognathic system. However, it should be kept in mind that during the masticatory cycle there is no equal excitation of all muscles at the same time (Hannam et al., 2008).

Another approach is to consider the biostatic equilibrium of the system, which is n-times statically indeterminate. In deformable body mechanics, several methods are available for solving statically indeterminate systems (the choice depends on the content of the task). In relation to the masticatory system, energy methods seem to be appropriate for analytical consideration. Analyses are often reduced to flat models on which symmetric loading only can be simulated (Kijak et al., 2015). Optimization algorithms are also used in the calculations to increase the number of equations or to reduce the number of unknowns (Nordin and Frankel, 2012). In these methods, the satisfaction of the static equilibrium and boundary conditions is obtained by adopting appropriate criteria such as the minimum of summed muscle forces, summed joint reaction, or summed elastic energies (Pruim et al., 1980; Koolstra and van Eijden, 1992; Osborn, 1995; Iwasaki et al., 2004; Schindler et al., 2007; Rues et al., 2008). In particular, the criteria of minimal energy, the minimal activation ratio, and the combination of minimal muscle force and moment moments are better reflected in the data obtained from *in vivo* measurements. Nevertheless, there is no consensus on the validity of applying them to the determination of muscle forces for treated structures (Zheng et al., 2019).

Despite the passing of a decade, the computer model types described in work (Hannam, 2011) are still valid and widely used in the analysis of craniofacial biomechanics. Many methods and models are used to consider muscle force identification, starting with the relatively simple static/quasi-static models, through dynamic models based on rigid body mechanics, to complex deformation models that use FEM formalism. Each of these solutions has its own application, justification and advantages in relation to specific research problems.

Numerical methods are commonly utilized in analyses of the biomechanics of the human jaw. Different approaches regarding the type of analysis (static/dynamic), the modeling of muscles and structures, and boundary conditions can be distinguished. In dynamic analyses, muscles are usually modeled as active elastic elements (Koolstra and Van Eijden, 2005). In static analyses, muscles can also have an active function, as forces are applied at the attachment sites (Korioth and Hannam, 1994; Reina et al., 2007). Another approach is to model muscles as elastic elements with a specific stiffness (Gross et al., 2001; Antic et al., 2015). This method involves loading the mandible with the bite force and then determining the forces in the muscles to balance it.

The most complex analyses include deformable models that illustrate changes in the structures under loading. Knowledge related to the analysis of the muscular system is now largely developed. The material characteristics of muscles, as well as their functioning, have been successfully described with appropriate mathematical functions and implemented in numerical analyses (Röhrle and Pullan, 2007; Böl and Reese, 2008; Röhrle et al., 2012; Weickenmeier et al., 2017). The first analysis, including the masseter muscle in a three-dimensional form, was performed by Röhrle and Pullan (2007). The results of their simulation, which was based on a combination of rigid-body (bone) and deformable-body (muscle), show the complex distribution of forces on the surface of the muscle attachment, as well as their variation with regards to the task and time of the simulation. Similar numerical calculations, still considering only the masseter muscle, can be found in the work of Weickenmeier et al. (2017). Their results were additionally combined with ultrasonographic measurements of changes in muscle volume. Such complex simulations require the determination of many parameters and variables, which are usually difficult to obtain and which are highly dependent on the case under consideration, i.e., fixed boundary conditions or the model’s geometry. Their results can therefore often be treated as highly individual.

In studies of the masticatory system, dynamic models are commonly utilized in simulations of mastication cycles and occlusion (Langenbach and Hannam, 1999; de Zee et al., 2007; Basafa et al., 2014; Dumitru et al., 2016; Marková and Gallo, 2016; Sagl et al., 2019). When determining parameters such as muscle forces, joint reactions, or moments, models based on rigid body mechanics often find their application (de Zee et al., 2007; Basafa et al., 2014; Marková and Gallo, 2016). The combination of rigid body dynamics with a deformable body model is primarily used (similarly to the aforementioned muscle structures) in analyses of deformations in the structures, e.g., the articular disk (Sagl et al., 2019). The basic division of dynamics models into forward (Langenbach and Hannam, 1999; Koolstra and Van Eijden, 2005; Martinez Choy et al., 2017) and inverse (de Zee et al., 2009; Marková and Gallo, 2016), which was presented in Buchanan et al. (2004), also seems correct. In forward-dynamics models, the movement of the mandible is forced by muscle work, the characteristics of which are introduced into the model. In inverse dynamics analyses, the input parameters are displacements and external loads, such as bite force. A description of each of these, along with the limitations and strengths, is presented in Buchanan et al. (2004). Moreover, a hybrid model is proposed, as it demonstrates significant accuracy of the results obtained with it. Another approach for identifying muscle forces is the biocybernetic model, in which muscles are treated as black boxes, with their properties adapting to external conditions (Kijak et al., 2015). This model does not require the introduction of a mathematical description of the muscle, or the characteristics of its work. It is based on the self-regulation of the system with different positions of the mandible, as well as the resulting changes in muscle length. Static and quasi-static models, based on the static equilibrium of the system, are considered to be the most basic. Moreover, they are arguably the easiest to develop (Schindler et al., 2007; Rues et al., 2008; Hannam, 2011).

The first analysis of the effect of macroscopic changes in mandibular geometry due to mandibular distraction was performed by de Zee et al. (2007). A model based on inverse dynamics and minimum muscle effort was used to determine muscle activity and forces in the masticatory system. The obtained results are characterized by a satisfactory correlation with the measurements of electrical muscle activity. Due to the lack of a direct, elementary relationship between muscle electric activity and muscle-generated force, a better correlation coefficient between the estimated and measured muscle activity is observed for static, isometric contraction case considerations (de Zee et al., 2007).

The main aim of this study was the analysis of changes in muscle forces (in terms of rigid body mechanics) resulting from single displacements of osteotomy segments following BSSO surgery. Additionally, based on three hypotheses, the results (muscle force values) obtained for individual cases were summed. They were then compared with the results obtained for the model in which the altered position of three osteotomy segments was introduced to the geometry of the model simultaneously. The presented work has a typically mechanical character, i.e., only force changes were considered, without taking into account biological changes. The analysis should be used to consider a hypothesis of the potential impact of bone geometry remodeling on changes in the biomechanics of the masticatory system. Moreover, the study could serve as a practical guide for surgeons as to which combination of changes may result in less favorable biomechanical conditions. The data for the study are comparative, i.e., case studies were carried out for identical boundary conditions.

## Materials and Methods

The presented work aimed to determine changes in muscle forces under static load conditions. The extreme case of mandible load, i.e., unilateral chewing, was considered (Korioth and Hannam, 1994; Choi et al., 2005). The adoption of the aforementioned loading scheme allowed for the analysis of the effect of geometry changes on the working and balancing sides. Mandibular loading involved the application of an bite force to the first molar on the right side, in which the line of action was perpendicular to the occlusal plane. This corresponded to the pattern presented in the work of Korioth and Hannam (1994).

**FIGURE 1:**
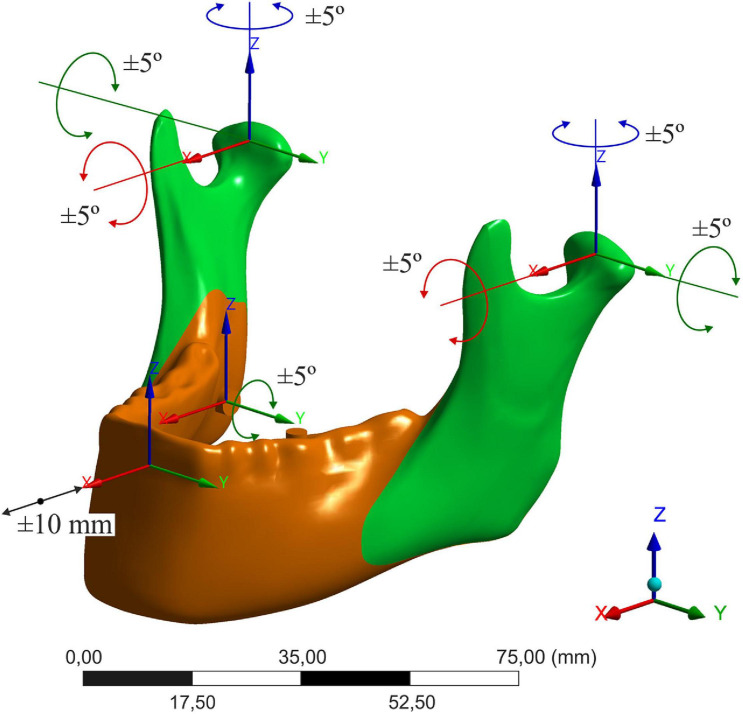
Considered displacements of bone segments.

Due to the fact that it is complex and difficult to predict the nature of the displacements, the discussion is limited to the five most commonly reported parameters that characterize changes during orthognathic surgery: distal segment translation in the sagittal axis (magnitude of advancement or setback), distal segment rotation in the sagittal plane, proximal segment pitch, yaw, and roll. For the same reason, the results for each displacement change were considered separately. The range of possible condyle rotations was 5° for all axes in both directions (Pachnicz and Ramos, 2021). The range of translation and rotation of the distal segment was 10 mm and 5°, respectively (Throckmorton et al., 1984; Van Sickels et al., 2002; Al-Moraissi and Wolford, 2016; Hasprayoon and Liao, 2020; Figure 1).

### Assumptions of the Numerical Model

The model is based on the basic assumptions of the statics and mechanics of a rigid body, which is in line with the information included in the paper of Hannam (2011). This type of model does not require the assignment of material properties, and therefore only the model’s geometry, constraints and loads are needed. The bone model was created from an anatomically correct polyurethane model of the human mandible (Synbone 8596), with mandibular elevator muscles being modeled as a spring with secant stiffness. The sites of the muscle attachment were determined based on anatomical atlases. The simulations were performed in the Ansys Workbench 16.0 program. Before starting the main analysis of changes in muscle force values, preliminary calculations were conducted in order to determine the muscle’s stiffness. This initial analysis was performed in two steps, which were conceptually similar to the forward and inward dynamic simulations:

Step I (forward): the boundary conditions for the unilateral clenching task were recreated according to data from Korioth and Hannam (1994). The model was loaded with muscle forces applied to the areas of their attachments, and the molar was first constrained for vertical translation. The value of the reaction force on the constrained tooth was obtained.

Step II (inward): the bite force (reaction force obtained in step I) was implemented to the model in the next step as an external load. The secant muscle stiffness was iteratively adjusted and determined so that at the given bite force (step 1) the value of the force in the muscle was consistent with the values adopted from the literature (Table 1).

**TABLE 1:** Muscle force values [N]: W, working side; B, balancing side.

**Masseter**				**Medial Pterygoid**		**Temporalis**					
**Superficial**		**Deep**				**Anterior**		**Middle**		**Posterior**	
**W**	**B**	**W**	**B**	**W**	**B**	**W**	**B**	**W**	**B**	**W**	**B**
137.1	114.2	58.8	49.0	146.8	104.9	115.3	91.6	63.1	64.1	44.6	29.5

The values presented in the work of Korioth and Hannam (1994) are given for the clenching tasks. Consequently, the contraction of the muscle can be assumed as quasi-isometric (Marková and Gallo, 2016), and there was therefore also a linear increase in bite force. Since the masticatory system is characterized by the fact that the response of the system (velocity, acceleration, forces, reactions, displacements) is correlated with the load (Stróżyk and Bałchanowski, 2018), the assumption of a constant value of secant stiffness (c) is justified.

With appropriate stiffness ratios, only the bite force will affect the value of the forces in the muscles. Scaling the stiffness value will only affect the model displacements. The reference stiffness, relative to which the others were defined, was determined for the superficial masseter. The calculations, based on the geometric dimensions of the muscle, were conducted using the elementary formula from the mechanics of deformable bodies (Eq. 1). The division of the elevator muscles, the cross-section area (PCSA), and the length of the masseter (l) were based on data from the literature (Van Eijden et al., 1997). The list of muscle stiffness is presented in Table 2.

**TABLE 2:** Stiffness of elastic elements c [N/mm].

	**Working side**	**Balancing side**
Masseter		
Superficial	11.00	9.43
Deep	12.50	10.71
Temporalis		
Anterior	12.50	9.93
Medial	7.80	7.92
Posterior	8.50	5.62
Medial pterygoid	21	15

(1) $$c=\frac{E\cdot P\,C\,S\,A}{l}$$

E- Young’s modulus (Gross et al., 2001), PCSA- muscle physiological cross-sectional area, l- length of the belly of the muscle.

### Numerical Simulation (Main Analysis)

In the calculations, the original model (after BSSO) was used, which consisted of the mandible (rigid) and elevator muscles (springs). The model was divided into distal and proximal segments by dissecting the split plane according to Obwegesser with Dal Pont modification osteotomy (Dal Pont, 1961). The positional changes of the segments were introduced at the level of geometry. A rigid connection was set between the segments to recreate the full bony union. The simulation was performed for 81 cases (10 rotations of the proximal segment in each of the three directions, for the working and balancing side, 10 angular and 10 translational changes for the distal segment).

**FIGURE 2:**
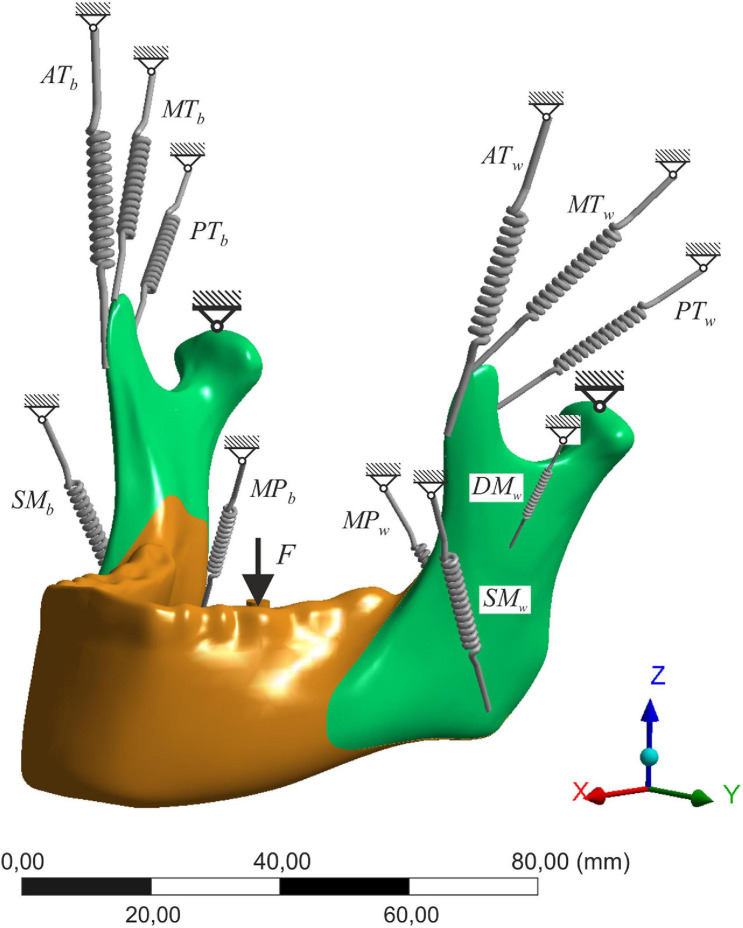
The scheme of the numerical model; SM, superficial masseter; DM, deep masseter; MP, medial pterygoid; AT, anterior temporalis; MT, medial temporalis; PT, posterior temporalis; w, working side; b, balancing side.

- The model was constrained in three degrees of freedom (only rotations allowed) in both condyles. The bite force was applied to the first molar (Figure 2).

## Results

For most of the considered displacements of osteotomy segments, the percentage changes in forces did not exceed, or were close to. 1%. In Table 3, the results for displacements where the percentage change exceeded 1.5% are shown. The results are given in comparison to the baseline model (state 0). The positive displacement values correspond to forward translation, counterclockwise rotation of the distal segment, outward yaw, lateral roll, and counterclockwise pitch rotations. Translation of the distal segment (DTr) has the greatest relevance for forces in the masticatory system. Significant differences occur in all muscles (−21.69 to 26.11%), with the lowest difference being in the medial pterygoid muscle (−16.31 to 7.01%), the position of which is altered during translation. Changes up to several percent can also be noticed for the rotation of the distal segment (DRot) in the sagittal plane. Only for single muscles and rotations of the proximal segment exceeding 3° did the difference in muscle force exceed 1.5%. Rotations of the proximal segment have the greatest impact on the deep masseter muscle. Additionally, differences of 1.62% (not included in the tables) are observed for almost all muscles for the highest Yaw rotation introduced on the working side.

**TABLE 3:** Percentage changes in the value of muscle forces when displacing the distal segment along the sagittal axis (DTr), the rotation of the distal segment along the transverse axis (DRot), the rotation of the proximal segment (Yaw, Roll, Pitch); S0- preoperative geometry model; abbreviations presented in Figure 2.

**DTr [mm]**	**SM_w_**	**DM_w_**	**MP_w_**	**AT_w_**	**MT_w_**	**PT_w_**	**SM_b_**	**DM_b_**	**MP_b_**	**AT_b_**	**MT_b_**	**PT_b_**
−10	–21.68	–21.69	–16.24	–21.67	–21.67	–21.67	–21.66	–21.65	–16.31	–21.67	–21.67	–21.66
−8	–17.61	–17.61	–12.55	–17.60	–17.60	–17.59	–17.59	–17.58	–12.60	–17.60	–17.60	–17.59
−6	–13.42	–13.43	–9.03	–13.42	–13.41	–13.41	–13.41	–13.40	–9.07	–13.41	–13.41	–13.41
−4	–9.11	–9.11	–5.74	–9.10	–9.10	–9.10	–9.10	–9.09	–5.76	–9.10	–9.10	–9.10
−2	–4.64	–4.64	–2.71	–4.64	–4.63	–4.63	–4.63	–4.63	–2.72	–4.63	–4.63	–4.63
0	0.00	0.00	0.00	0.00	0.00	0.00	0.00	0.00	0.00	0.00	0.00	0.00
2	4.82	4.82	2.34	4.82	4.82	4.82	4.82	4.82	2.35	4.82	4.82	4.82
4	9.84	9.85	4.25	9.84	9.84	9.83	9.84	9.83	4.28	9.84	9.84	9.83
6	15.07	15.07	5.69	15.06	15.06	15.05	15.06	15.05	5.74	15.06	15.06	15.05
8	20.49	20.49	6.60	20.48	20.48	20.47	20.48	20.46	6.66	20.48	20.48	20.47
10	26.11	26.11	6.94	26.10	26.09	26.09	26.10	26.08	7.01	26.10	26.10	26.09

**DRot [o]**	**SMw**	**DMw**	**MPw**	**ATw**	**MTw**	**PTw**	**SMb**	**DMb**	**MPb**	**ATb**	**MTb**	**PTb**

−5	–5.60	–5.61	–5.24	–5.59	–5.59	–5.59	–5.59	–5.59	–5.27	–5.59	–5.59	–5.59
−4	–4.36	–4.36	–4.03	–4.35	–4.35	–4.35	–4.35	–4.35	–4.06	–4.35	–4.35	–4.35
−3	–3.17	–3.18	–2.90	–3.17	–3.17	–3.16	–3.17	–3.17	–2.92	–3.17	–3.17	–3.17
−2	–2.05	–2.05	–1.85	–2.05	–2.05	–2.05	–2.05	–2.05	–1.87	–2.05	–2.05	–2.05
−1	–0.99	–0.99	–0.89	–0.99	–0.99	–0.99	–0.99	–0.99	–0.89	–0.99	–0.99	–0.99
0	0.00	0.00	0.00	0.00	0.00	0.00	0.00	0.00	0.00	0.00	0.00	0.00
1	0.93	0.93	0.80	0.93	0.93	0.93	0.93	0.93	0.81	0.93	0.93	0.93
2	1.79	1.79	1.52	1.79	1.79	1.79	1.79	1.79	1.53	1.79	1.79	1.79
3	2.59	2.59	2.15	2.58	2.58	2.58	2.58	2.58	2.16	2.58	2.58	2.59
4	3.31	3.32	2.69	3.31	3.30	3.30	3.31	3.31	2.71	3.31	3.31	3.31
5	3.97	3.98	3.15	3.97	3.96	3.96	3.96	3.97	3.17	3.97	3.97	3.97
												

	**DM_w_**			**MT_w_**	**PT_w_**	**DM_b_**			**PT_b_**
	**Yaw_w_**	**Roll_w_**	**Pitch_w_**	**Roll_w_**	**Pitch_w_**	**Yaw_b_**	**Roll_b_**	**Pitch_b_**	**Pitch_b_**

−5	–1.70	–3.21	2.99	1.82	–2.96	–1.93	–3.31	2.76	–3.18
−4		–2.57	2.43		–2.37	–1.56	–2.64	2.26	–2.54
−3		–1.92	1.86		–1.77		–1.97	1.73	–1.90
0									
3		1.92	–2.07		1.78		1.95	–1.97	1.88
4	1.51	2.56	–2.82		2.37	1.65	2.59	–2.69	2.50
5	1.90	3.20	–3.59		2.97	2.08	3.24	–3.44	3.13

In addition, a preliminary analysis of muscle forces for complex geometrical changes was carried out based on the results obtained for single geometry modifications. The calculations were performed for 10 quasi-randomly selected assemblies: five planned assemblies (contact between segments) and five random displacements (Supplementary Table 4). The purpose of the analysis was to assess whether it is possible to infer changes in muscle forces for complex cases by comparing simple cases. The method adopted summed the differences (ΣDiff) between a particular displacement and state 0, which was then added to the values for state 0. The percentage differences between the values obtained for the model with complex displacements and those obtained for the estimated ones reach a maximum of 1.2%. The approximation accuracy for most muscles was above 99.9%.

## Discussion

The paper presents the determination of muscle strength concerned issues based on the mechanics of a rigid body. A spatial model was used for the calculations, and the action of the mandibular elevator muscles was taken into account. A new method of determining muscle stiffness was utilized, which can be used in further numerical simulations.

A clear relationship between the morphology of the facial bones and the muscular system has been observed (Van Spronsen et al., 1997; Sella-Tunis et al., 2018). As a result of orthognathic procedures, bone geometry is altered, which affects, among other things, muscle position and muscle length, thereby altering the biomechanical conditions (Jung et al., 2015). These changes cause the remodeling of the musculoskeletal system (Grunheid et al., 2009), and are also a factor in the remodeling process of the bones themselves (Verhelst et al., 2019; Yamada et al., 2020). The analysis of changes in muscle forces resulting from changes in geometry is therefore an important issue, e.g., when considering temporomandibular joint loading. It can be used to illustrate new biomechanical conditions, and also plays a role in the monitoring of system abnormalities (Kijak et al., 2015).

Preliminary, and therefore simplified considerations, concerning the impact of orthognathic surgery on the biomechanics of the mandible can be found in the literature. Throckmorton et al. (1984) limited their analysis to a flat, symmetrical loading model and two muscles: the masseter and the temporal. Geometry changes included changes in the occlusal plane angle (maxilla reposition), gonial angle and distal segment translation. Their effect was determined by the ratio of each muscle’s moment arm to the moment of the occlusal force (Throckmorton et al., 1980). These types of results, as was noted by the authors, are only of an illustrative nature in the case of remodeling muscles, as well as in the case of changes in their motoric properties, which was also mentioned in papers (Dicker et al., 2007, 2015; Grunheid et al., 2009). Dicker et al. (2007, 2012) noticed significant changes in muscle directions, as well as a reduction in the size of the elevator muscles after mandible advancement surgery. However, no further correlation between these parameters and later changes in TMJ or occlusion force can be indicated. Hwang et al. (2000), however, suggested a possible increase in joint loading, which in turn leads to subsequent condylar resorption. The results of the presented study, as with the conclusions drawn by Dicker et al. (2007), do not support the hypothesis explaining the reduction in muscle size due to the improvement of biomechanical conditions in the correction of mandibular retrognathia. Distal segment advancement has the greatest impact on changes of muscle forces. A translation of only 4 mm results in an increase in all (except for pterygoids) muscle strength by almost 10% (Table 3). The distal segment displacement is also associated with a change in the position of the medial pterygoid muscle. This could suggest that the greatest changes in force values will occur in it. However, our results show the opposite trend. Moreover, changing its position will affect the entire muscular system due to the synergy between the muscles (Herring, 2007).

Although the identification of muscle forces has been carried out for several decades, it is rarely was considered when studying the effects of craniofacial geometry or its changes. Few examples of this type of analysis can be found in the literature from recent years. The first analysis of macroscopic changes in mandibular geometry on musculoskeletal mechanics using a 3D rigid-body model was performed by de Zee et al. (2007). The study aimed to validate the model by comparing the measures of the EMG activity of muscles with the results obtained in the corresponding computer model. The activity was determined for different cases of jaw clenching and dynamic tasks. The determination of forces acting on the masticatory organ from EMG measurements is not entirely correct for dynamic analysis. A more accurate, linear relationship between EMG readings and forces in the muscles can be observed for cases of isometric contraction (de Zee et al., 2007). Confirmation of this thesis can also be observed in other publications (Buchanan et al., 2004).

The same model has also been used to analyze muscle and joint reaction forces for different angles of the anterior fossa slope (Marková and Gallo, 2016). In the given publication, the comparison of forces was performed for two cases of empty chewing and unilateral clenching. The authors noted that the value of muscle forces not only depends on the magnitude of muscle activation, but also on the moment arms of the lines of action, which are affected by the slope inclination. In the paper (Zheng et al., 2019), the inverse identification of muscle forces was performed. This method uses an optimization algorithm to determine force values before and after surgery based on the size of cross sections obtained from CT images. It gives accurate results, but is only suitable for considering individual cases.

The effect of changes in mandibular geometry resulting from BSSO surgery on the muscular system has not previously been considered on a three-dimensional model. The mentioned works of Throckmorton et al. (1984) and Dicker et al. (2012) limit the consideration to a planar system. However, in the literature there are analyses in which the influence of changes in bone geometry is considered. The papers by Shu et al. (2020) and Shu et al. (2019) examined changes in TMJ loading following the correction of a prognathism. Both of these papers compared the results of simulations performed for a model before and after treatment. The models were loaded by applying forces to the areas corresponding to the muscle attachments. Considering the results obtained in our work, as well as similar analyses of muscle force changes, it is visible that the authors used the same muscle forces for the pre- and post-treatment model in their work. However, a more accurate analysis would require adjusting the muscle forces to the new conditions. A different way of loading the models with pre- and post-treatment geometry is presented in Xiangdong et al. (2012). The mandible is loaded by introducing preload in truss-type elements in order to simulate the mandibular opening and closing muscles. The authors do not include, however, a substantive justification for the applied loading, and only provide maximum values for neutral conditions. They also note that an increased accuracy of the simulation results can be achieved by properly selecting muscle forces that are specific for the patient.

Compared to the models that are currently used, the one presented in this paper is an elementary model based on the substantive principles of rigid body mechanics – it is effective for the purpose of this paper. Rigid models are commonly utilized in muscle force analyses. Elements of deformable body mechanics are introduced when considering changes of the strain and stress field. In the load case adopted for the analysis (clenching), displacements occur, albeit small in relation to the cross-sectional dimensions of the mandible. They result from the positioning of the condyles in the fossa (Marková and Gallo, 2016). With the assumption that there is no displacement in both condyles (constrained in three degrees of freedom- translations), but allowing for minor rotations, the model can still be considered as a static one.

The model presented in this paper also has its economic justification due to the number of considered cases (80) of single geometry alterations. Complex models usually require significant computation times. Therefore, when possible, the use of a simple model construction and the simultaneous correct representation of the considered phenomenon is recommended.

The results obtained from the static model could be used in a simple and direct way for verifying hypotheses concerning the impact of complex changes of geometry. This is achieved by assembling the values obtained for individual cases. In analyses based on dynamic models, results are obtained in the form of force diagrams from which maximum values can be read. They could therefore be used to analyze changes during different tasks, e.g., the chewing cycle.

Rotations of the proximal segments affect mandible biomechanics to a much lesser extent. The percentage of change is almost the same for all muscles on the mandible side with no geometry correction. On the side where rotations were introduced, the differences depend on the muscle and the type of displacement. Yaw rotation has the opposite effect on the superficial masseter muscle and the medial pterygoid muscle when compared to the rest of the muscles. Forces decrease with an increasing angle (outward rotation). Inward tilting, however, causes an increase in force values. It also results in the opposite nature of changes in the two parts of the masseter muscle. The same effect for all muscles, except the masseter muscle, is observed for displacements in the frontal plane (Roll). This rotation has the greatest impact on the deep head of the masseter muscle. Pitch rotation, as with yaw, has the opposite effect on the two masseter muscle heads. The force in the superficial masseter muscle, contrary to the deep masseter muscle, increases with counterclockwise rotation. Percentage changes in the forces in the deep masseter muscle are the greatest and range from 3.44 to 2.76%. The contrasting nature of changes is also observed within the temporal muscle. The vast majority of differences in muscle force values do not exceed 1% for the maximum considered ranges of rotation, i.e., 5°.

The authors have attempted to estimate force values in complex, displacement assembly cases based on results obtained from singular cases. The principle of superposition does not apply because the boundary conditions change along with the geometry. The resultant vector applies to a very small extent due to the lack of geometric models overlapping, and does not coincide with the direction of the muscle force vector in the assembly model. The proposed practical approach involves the summing of the differences in the muscle force values from the single displacements and state zero, which was then added to the values for state 0. In each of the considered cases, the calculated value differed from the value obtained from the simulation by less than 0.5%. In most of the displacement combinations, this method allowed the value of muscle strength to be estimated with an accuracy exceeding, or close to, 99%. Although only verified in a small number of cases, this method may be of practical use. On its basis, it can be concluded that appropriate changes in the mandible geometry will intensify the impact on muscle forces (e.g., clockwise pitch rotation in mandible advancement), and other changes will in turn level each other (e.g., counterclockwise pitch in mandible setback). The results can be used, among others, as a guideline for surgeons, which provide suggestions about which simple cases should be avoided. They may also be utilized for scaling muscle forces in numerical analyses of the masticatory system after BSSO treatment. Tables with results that can be used for individual calculations are included in the Supplementary Material (Supplementary Tables 1–3).

The limitations of the considerations carried out in this work should be noted. The model adopted for the calculations is a static one that simulates the conditions of isometric contraction for a specific position of the jaw. Full characterization of the muscle forces can be obtained from dynamic systems, the determination of which can additionally be based on the work criterion, which is impossible in a static system. Such models, however, require the introduction of accurate characteristics regarding muscle work, as well as mandibular displacement and loading of the dental arch (Peck et al., 2000; Koolstra, 2002; Stróżyk and Bałchanowski, 2018). The results presented in the given study are calculated for the assumed rate of displacements (translation every 2 mm, rotation every 1°). Nevertheless, the changes are continuous. Moreover, only 10 cases of displacement assemblies were analyzed in the paper. However, the number of possible combinations of the relative positions of the osteotomy segments is unlimited. The position of the distal segment is planned, and its displacements are pre-determined. The final position of the proximal segments, however, is partly a result of both the new position of the distal segment and the factors related to the surgical intervention (condylar sagging, osteotomy methods, line of fracture). Their exact position after the surgery is therefore complicated to predict (Pachnicz and Ramos, 2021). It should be noted that the method proposed in this paper for estimating changes in cases of complex displacements is an approximate method for practical applications. In the analyzed cases it allowed results to be obtained that were close to the expected ones. Further work should consider verification of the method for a larger number of cases, preferably based on medical statistics. It will also be interesting to develop other hypotheses that would be based, e.g., on the minimum energy of the system, which will in turn allow single cases to be summed.

## Conclusion

This study shows, from a mechanical point of view, that changes in the geometry of the mandible due to BSSO surgery will influence muscle force values. The displacement of the distal segment has the greatest influence on force differences, while the rotation of the proximal segments has the least influence. At this stage of the study, it can be concluded that the muscle forces for a complex displacement case can be estimated with high agreement by summing the differences of single cases with condition 0.

## Data Availability Statement

The original contributions presented in the study are included in the article/Supplementary Material, further inquiries can be directed to the corresponding author.

## Author Contributions

DP and PS contributed to conception, design, drafted, and critically revised the manuscript. Both authors gave their final approval and agree to be accountable for all aspects of the work.

## Conflict of Interest

The authors declare that the research was conducted in the absence of any commercial or financial relationships that could be construed as a potential conflict of interest.
