# Supplementary material for: A Biomechanical Analysis of Muscle Force Changes After Bilateral Sagittal Split Osteotomy
Source: Front Physiol. 2021 Jun 3;12:679644. doi: 10.3389/fphys.2021.679644 (PMC8209381; doi:10.3389/fphys.2021.679644)
Supplement: Supplementary file 1 [file Table_1.docx]

Supplementary Table 1. Muscle force values (10 N first molar loading) for distal segment displacements: Tr- translation, Rot- rotation; SM-superficial masseter, DM- deep masseter, MP- medial pterygoid, AT- anterior temporalis, MT-medial temporalis, PT- posterior temporalis

| DTr  [mm] | SM_w_ | DM_w_ | MP_w_ | AT_w_ | MT_w_ | PT_w_ | SM_b_ | DM_b_ | MP_b_ | AT_b_ | MT_b_ | PT_b_ |
| --- | --- | --- | --- | --- | --- | --- | --- | --- | --- | --- | --- | --- |
| -10 | 2,075 | 0,881 | 2,265 | 1,797 | 0,981 | 0,633 | 1,778 | 0,754 | 1,618 | 1,427 | 0,996 | 0,419 |
| -8 | 2,183 | 0,926 | 2,366 | 1,891 | 1,032 | 0,666 | 1,870 | 0,793 | 1,689 | 1,501 | 1,048 | 0,441 |
| -6 | 2,294 | 0,973 | 2,461 | 1,987 | 1,084 | 0,700 | 1,965 | 0,833 | 1,758 | 1,577 | 1,101 | 0,463 |
| -4 | 2,408 | 1,022 | 2,551 | 2,085 | 1,138 | 0,735 | 2,063 | 0,875 | 1,822 | 1,656 | 1,155 | 0,486 |
| -2 | 2,526 | 1,072 | 2,633 | 2,188 | 1,194 | 0,771 | 2,164 | 0,918 | 1,881 | 1,737 | 1,212 | 0,510 |
| 0 | 2,648 | 1,124 | 2,707 | 2,294 | 1,252 | 0,808 | 2,268 | 0,962 | 1,934 | 1,821 | 1,271 | 0,534 |
| 2 | 2,776 | 1,178 | 2,771 | 2,404 | 1,312 | 0,847 | 2,377 | 1,008 | 1,980 | 1,908 | 1,332 | 0,560 |
| 4 | 2,908 | 1,235 | 2,823 | 2,518 | 1,375 | 0,887 | 2,491 | 1,056 | 2,018 | 1,999 | 1,395 | 0,586 |
| 6 | 3,046 | 1,293 | 2,863 | 2,638 | 1,440 | 0,929 | 2,609 | 1,106 | 2,047 | 2,094 | 1,461 | 0,614 |
| 8 | 3,189 | 1,354 | 2,889 | 2,761 | 1,507 | 0,973 | 2,731 | 1,158 | 2,066 | 2,192 | 1,530 | 0,643 |
| 10 | 3,337 | 1,417 | 2,900 | 2,890 | 1,577 | 1,018 | 2,858 | 1,212 | 2,074 | 2,294 | 1,601 | 0,673 |
| DRot [^o^] | SM_w_ | DM_w_ | MP_w_ | AT_w_ | MT_w_ | PT_w_ | SM_b_ | DM_b_ | MP_b_ | AT_b_ | MT_b_ | PT_b_ |
| -5 | 2,580 | 1,095 | 2,643 | 2,235 | 1,220 | 0,787 | 2,210 | 0,937 | 1,888 | 1,774 | 1,238 | 0,520 |
| -4 | 2,595 | 1,101 | 2,657 | 2,247 | 1,227 | 0,792 | 2,223 | 0,943 | 1,898 | 1,784 | 1,245 | 0,523 |
| -3 | 2,609 | 1,107 | 2,670 | 2,259 | 1,233 | 0,796 | 2,235 | 0,948 | 1,908 | 1,794 | 1,252 | 0,526 |
| -2 | 2,622 | 1,113 | 2,683 | 2,271 | 1,240 | 0,800 | 2,246 | 0,953 | 1,917 | 1,803 | 1,258 | 0,529 |
| -1 | 2,636 | 1,119 | 2,695 | 2,282 | 1,246 | 0,804 | 2,258 | 0,957 | 1,926 | 1,812 | 1,265 | 0,532 |
| 0 | 2,648 | 1,124 | 2,707 | 2,294 | 1,252 | 0,808 | 2,268 | 0,962 | 1,934 | 1,821 | 1,271 | 0,534 |
| 1 | 2,661 | 1,129 | 2,718 | 2,304 | 1,258 | 0,812 | 2,279 | 0,967 | 1,942 | 1,829 | 1,277 | 0,537 |
| 2 | 2,673 | 1,134 | 2,728 | 2,315 | 1,263 | 0,815 | 2,289 | 0,971 | 1,949 | 1,838 | 1,282 | 0,539 |
| 3 | 2,684 | 1,139 | 2,738 | 2,324 | 1,269 | 0,819 | 2,299 | 0,975 | 1,956 | 1,845 | 1,288 | 0,541 |
| 4 | 2,695 | 1,144 | 2,747 | 2,334 | 1,274 | 0,822 | 2,308 | 0,979 | 1,963 | 1,853 | 1,293 | 0,544 |
| 5 | 2,706 | 1,149 | 2,756 | 2,343 | 1,279 | 0,826 | 2,317 | 0,983 | 1,969 | 1,860 | 1,298 | 0,546 |
