# Supplementary material for: A Biomechanical Analysis of Muscle Force Changes After Bilateral Sagittal Split Osteotomy
Source: Front Physiol. 2021 Jun 3;12:679644. doi: 10.3389/fphys.2021.679644 (PMC8209381; doi:10.3389/fphys.2021.679644)
Supplement: Supplementary file 2 [file Table_2.docx]

Supplementary Table 2. Muscle force values (10 N first molar loading) for rotations of the proximal segment on working side

| Yaw_w_ [^o^] | SM_w_ | | DM_w_ | | MP_w_ | | AT_w_ | MT_w_ | PT_w_ | SM_b_ | DM_b_ | MP_b_ | AT_b_ | MT_b_ | PT_b_ |
| --- | --- | --- | --- | --- | --- | --- | --- | --- | --- | --- | --- | --- | --- | --- | --- |
| -5 | 2,672 | | 1,105 | | 2,750 | | 2,270 | 1,236 | 0,801 | 2,305 | 0,978 | 1,965 | 1,850 | 1,291 | 0,543 |
| -4 | 2,667 | | 1,109 | | 2,741 | | 2,275 | 1,240 | 0,803 | 2,297 | 0,974 | 1,959 | 1,844 | 1,287 | 0,541 |
| -3 | 2,662 | | 1,112 | | 2,732 | | 2,281 | 1,243 | 0,804 | 2,289 | 0,971 | 1,952 | 1,838 | 1,282 | 0,539 |
| -2 | 2,657 | | 1,116 | | 2,723 | | 2,286 | 1,246 | 0,806 | 2,282 | 0,968 | 1,946 | 1,832 | 1,278 | 0,537 |
| -1 | 2,653 | | 1,120 | | 2,715 | | 2,290 | 1,249 | 0,807 | 2,275 | 0,965 | 1,940 | 1,826 | 1,274 | 0,536 |
| 0 | 2,648 | | 1,124 | | 2,707 | | 2,294 | 1,252 | 0,808 | 2,268 | 0,962 | 1,934 | 1,821 | 1,271 | 0,534 |
| 1 | 2,644 | | 1,128 | | 2,699 | | 2,297 | 1,255 | 0,809 | 2,262 | 0,959 | 1,929 | 1,816 | 1,267 | 0,533 |
| 2 | 2,640 | | 1,132 | | 2,692 | | 2,300 | 1,257 | 0,810 | 2,256 | 0,957 | 1,924 | 1,811 | 1,264 | 0,531 |
| 3 | 2,637 | | 1,137 | | 2,686 | | 2,302 | 1,260 | 0,812 | 2,251 | 0,955 | 1,919 | 1,807 | 1,261 | 0,530 |
| 4 | 2,633 | | 1,141 | | 2,680 | | 2,304 | 1,262 | 0,813 | 2,246 | 0,953 | 1,915 | 1,803 | 1,258 | 0,529 |
| 5 | 2,630 | | 1,145 | | 2,674 | | 2,305 | 1,264 | 0,814 | 2,241 | 0,951 | 1,911 | 1,799 | 1,256 | 0,528 |
| Roll_w_ [^o^] | SM_w_ | | DM_w_ | | MP_w_ | | AT_w_ | MT_w_ | PT_w_ | SM_b_ | DM_b_ | MP_b_ | AT_b_ | MT_b_ | PT_b_ |
| -5 | 2,622 | | 1,088 | | 2,725 | | 2,313 | 1,274 | 0,819 | 2,283 | 0,968 | 1,947 | 1,833 | 1,279 | 0,538 |
| -4 | 2,628 | | 1,095 | | 2,720 | | 2,308 | 1,270 | 0,817 | 2,280 | 0,967 | 1,944 | 1,830 | 1,277 | 0,537 |
| -3 | 2,634 | | 1,103 | | 2,716 | | 2,304 | 1,265 | 0,814 | 2,276 | 0,965 | 1,941 | 1,827 | 1,275 | 0,536 |
| -2 | 2,640 | | 1,110 | | 2,713 | | 2,300 | 1,261 | 0,812 | 2,273 | 0,964 | 1,938 | 1,825 | 1,274 | 0,535 |
| -1 | 2,644 | | 1,117 | | 2,709 | | 2,296 | 1,256 | 0,810 | 2,271 | 0,963 | 1,936 | 1,823 | 1,272 | 0,535 |
| 0 | 2,648 | | 1,124 | | 2,707 | | 2,294 | 1,252 | 0,808 | 2,268 | 0,962 | 1,934 | 1,821 | 1,271 | 0,534 |
| 1 | 2,652 | | 1,131 | | 2,705 | | 2,291 | 1,248 | 0,806 | 2,267 | 0,961 | 1,932 | 1,819 | 1,270 | 0,534 |
| 2 | 2,655 | | 1,138 | | 2,703 | | 2,288 | 1,244 | 0,804 | 2,265 | 0,961 | 1,931 | 1,818 | 1,269 | 0,533 |
| 3 | 2,657 | | 1,146 | | 2,701 | | 2,286 | 1,240 | 0,803 | 2,264 | 0,960 | 1,930 | 1,817 | 1,268 | 0,533 |
| 4 | 2,659 | | 1,153 | | 2,700 | | 2,285 | 1,236 | 0,801 | 2,263 | 0,960 | 1,929 | 1,817 | 1,268 | 0,533 |
| 5 | 2,660 | | 1,160 | | 2,700 | | 2,284 | 1,233 | 0,800 | 2,263 | 0,960 | 1,929 | 1,816 | 1,268 | 0,533 |
| Pitch_w_ [^o^] | | SM_w_ | | DM_w_ | | MP_w_ | AT_w_ | MT_w_ | PT_w_ | SM_b_ | DM_b_ | MP_b_ | AT_b_ | MT_b_ | PT_b_ |
| -5 | | 2,633 | | 1,157 | | 2,743 | 2,296 | 1,250 | 0,784 | 2,299 | 0,975 | 1,960 | 1,845 | 1,288 | 0,541 |
| -4 | | 2,636 | | 1,151 | | 2,735 | 2,296 | 1,251 | 0,789 | 2,292 | 0,972 | 1,954 | 1,840 | 1,284 | 0,540 |
| -3 | | 2,639 | | 1,145 | | 2,727 | 2,296 | 1,251 | 0,794 | 2,285 | 0,969 | 1,948 | 1,834 | 1,280 | 0,538 |
| -2 | | 2,642 | | 1,138 | | 2,720 | 2,295 | 1,251 | 0,799 | 2,279 | 0,967 | 1,943 | 1,830 | 1,277 | 0,537 |
| -1 | | 2,645 | | 1,131 | | 2,713 | 2,294 | 1,252 | 0,803 | 2,274 | 0,964 | 1,938 | 1,825 | 1,274 | 0,535 |
| 0 | | 2,648 | | 1,124 | | 2,707 | 2,294 | 1,252 | 0,808 | 2,268 | 0,962 | 1,934 | 1,821 | 1,271 | 0,534 |
| 1 | | 2,652 | | 1,117 | | 2,701 | 2,292 | 1,252 | 0,813 | 2,264 | 0,960 | 1,930 | 1,817 | 1,268 | 0,533 |
| 2 | | 2,655 | | 1,109 | | 2,696 | 2,291 | 1,252 | 0,818 | 2,259 | 0,958 | 1,926 | 1,814 | 1,266 | 0,532 |
| 3 | | 2,658 | | 1,101 | | 2,691 | 2,290 | 1,252 | 0,822 | 2,255 | 0,957 | 1,923 | 1,810 | 1,263 | 0,531 |
| 4 | | 2,662 | | 1,093 | | 2,687 | 2,288 | 1,252 | 0,827 | 2,252 | 0,955 | 1,920 | 1,808 | 1,262 | 0,530 |
| 5 | | 2,665 | | 1,084 | | 2,684 | 2,286 | 1,251 | 0,832 | 2,249 | 0,954 | 1,917 | 1,805 | 1,260 | 0,530 |
