# Supplementary material for: A Biomechanical Analysis of Muscle Force Changes After Bilateral Sagittal Split Osteotomy
Source: Front Physiol. 2021 Jun 3;12:679644. doi: 10.3389/fphys.2021.679644 (PMC8209381; doi:10.3389/fphys.2021.679644)
Supplement: Supplementary file 3 [file Table_3.docx]

Supplementary Table 3. Muscle force values (10 N first molar loading) for rotations of the proximal segment on balancing side

| Yaw_b_ [^o^] | | SM_w_ | | | DM_w_ | | | MP_w_ | | | AT_w_ | | | MT_w_ | | | PT_w_ | | | SM_b_ | | | DM_b_ | | | MP_b_ | | | AT_b_ | | | MT_b_ | | PT_b_ | |
| --- | --- | --- | --- | --- | --- | --- | --- | --- | --- | --- | --- | --- | --- | --- | --- | --- | --- | --- | --- | --- | --- | --- | --- | --- | --- | --- | --- | --- | --- | --- | --- | --- | --- | --- | --- |
| -5 | | 2,685 | | | 1,139 | | | 2,744 | | | 2,325 | | | 1,269 | | | 0,819 | | | 2,283 | | | 0,943 | | | 1,960 | | | 1,798 | | | 1,252 | | 0,528 | |
| -4 | | 2,677 | | | 1,136 | | | 2,736 | | | 2,318 | | | 1,265 | | | 0,817 | | | 2,280 | | | 0,947 | | | 1,955 | | | 1,803 | | | 1,256 | | 0,530 | |
| -3 | | 2,669 | | | 1,133 | | | 2,728 | | | 2,311 | | | 1,262 | | | 0,814 | | | 2,277 | | | 0,951 | | | 1,949 | | | 1,808 | | | 1,260 | | 0,531 | |
| -2 | | 2,662 | | | 1,130 | | | 2,721 | | | 2,305 | | | 1,258 | | | 0,812 | | | 2,274 | | | 0,954 | | | 1,944 | | | 1,813 | | | 1,264 | | 0,532 | |
| -1 | | 2,655 | | | 1,127 | | | 2,713 | | | 2,299 | | | 1,255 | | | 0,810 | | | 2,271 | | | 0,958 | | | 1,939 | | | 1,817 | | | 1,267 | | 0,533 | |
| 0 | | 2,648 | | | 1,124 | | | 2,707 | | | 2,294 | | | 1,252 | | | 0,808 | | | 2,268 | | | 0,962 | | | 1,934 | | | 1,821 | | | 1,271 | | 0,534 | |
| 1 | | 2,642 | | | 1,122 | | | 2,700 | | | 2,288 | | | 1,249 | | | 0,806 | | | 2,266 | | | 0,966 | | | 1,929 | | | 1,824 | | | 1,274 | | 0,535 | |
| 2 | | 2,637 | | | 1,119 | | | 2,694 | | | 2,283 | | | 1,246 | | | 0,804 | | | 2,263 | | | 0,970 | | | 1,925 | | | 1,827 | | | 1,277 | | 0,536 | |
| 3 | | 2,631 | | | 1,117 | | | 2,689 | | | 2,278 | | | 1,244 | | | 0,803 | | | 2,261 | | | 0,974 | | | 1,921 | | | 1,830 | | | 1,280 | | 0,537 | |
| 4 | | 2,626 | | | 1,115 | | | 2,684 | | | 2,274 | | | 1,241 | | | 0,801 | | | 2,258 | | | 0,978 | | | 1,918 | | | 1,832 | | | 1,283 | | 0,538 | |
| 5 | | 2,622 | | | 1,113 | | | 2,679 | | | 2,270 | | | 1,239 | | | 0,800 | | | 2,256 | | | 0,982 | | | 1,914 | | | 1,833 | | | 1,285 | | 0,539 | |
| Roll_b_ [^o^] | SM_w_ | | | DM_w_ | | | MP_w_ | | | AT_w_ | | | MT_w_ | | | PT_w_ | | | SM_b_ | | | DM_b_ | | | MP_b_ | | | AT_b_ | | | MT_b_ | | | PT_b_ | |
| -5 | 2,663 | | | 1,130 | | | 2,722 | | | 2,306 | | | 1,259 | | | 0,813 | | | 2,243 | | | 0,930 | | | 1,944 | | | 1,834 | | | 1,292 | | | 0,541 | |
| -4 | 2,659 | | | 1,129 | | | 2,718 | | | 2,303 | | | 1,257 | | | 0,811 | | | 2,249 | | | 0,937 | | | 1,942 | | | 1,831 | | | 1,288 | | | 0,539 | |
| -3 | 2,656 | | | 1,127 | | | 2,715 | | | 2,300 | | | 1,255 | | | 0,810 | | | 2,255 | | | 0,943 | | | 1,939 | | | 1,828 | | | 1,283 | | | 0,538 | |
| -2 | 2,653 | | | 1,126 | | | 2,712 | | | 2,298 | | | 1,254 | | | 0,810 | | | 2,260 | | | 0,949 | | | 1,937 | | | 1,825 | | | 1,279 | | | 0,537 | |
| -1 | 2,651 | | | 1,125 | | | 2,709 | | | 2,295 | | | 1,253 | | | 0,809 | | | 2,265 | | | 0,956 | | | 1,936 | | | 1,823 | | | 1,275 | | | 0,535 | |
| 0 | 2,648 | | | 1,124 | | | 2,707 | | | 2,294 | | | 1,252 | | | 0,808 | | | 2,268 | | | 0,962 | | | 1,934 | | | 1,821 | | | 1,271 | | | 0,534 | |
| 1 | 2,647 | | | 1,123 | | | 2,705 | | | 2,292 | | | 1,251 | | | 0,808 | | | 2,272 | | | 0,968 | | | 1,933 | | | 1,819 | | | 1,267 | | | 0,533 | |
| 2 | 2,645 | | | 1,123 | | | 2,703 | | | 2,291 | | | 1,250 | | | 0,807 | | | 2,275 | | | 0,975 | | | 1,932 | | | 1,817 | | | 1,263 | | | 0,532 | |
| 3 | 2,644 | | | 1,122 | | | 2,702 | | | 2,290 | | | 1,250 | | | 0,807 | | | 2,277 | | | 0,981 | | | 1,931 | | | 1,816 | | | 1,259 | | | 0,531 | |
| 4 | 2,643 | | | 1,122 | | | 2,702 | | | 2,289 | | | 1,249 | | | 0,807 | | | 2,278 | | | 0,987 | | | 1,930 | | | 1,815 | | | 1,256 | | | 0,530 | |
| 5 | 2,643 | | | 1,122 | | | 2,701 | | | 2,289 | | | 1,249 | | | 0,806 | | | 2,280 | | | 0,993 | | | 1,930 | | | 1,814 | | | 1,252 | | | 0,529 | |
| Pitch_b_ [^o^] | | | SM_w_ | | | DM_w_ | | | MP_w_ | | | AT_w_ | | | MT_w_ | | | PT_w_ | | | SM_b_ | | | DM_b_ | | | MP_b_ | | | AT_b_ | | | MT_b_ | | PT_b_ |
| -5 | | | 2,678 | | | 1,137 | | | 2,737 | | | 2,319 | | | 1,266 | | | 0,817 | | | 2,250 | | | 0,988 | | | 1,955 | | | 1,819 | | | 1,266 | | 0,517 |
| -4 | | | 2,671 | | | 1,134 | | | 2,730 | | | 2,313 | | | 1,263 | | | 0,815 | | | 2,254 | | | 0,984 | | | 1,951 | | | 1,820 | | | 1,267 | | 0,521 |
| -3 | | | 2,665 | | | 1,131 | | | 2,724 | | | 2,308 | | | 1,260 | | | 0,813 | | | 2,258 | | | 0,979 | | | 1,946 | | | 1,820 | | | 1,268 | | 0,524 |
| -2 | | | 2,659 | | | 1,129 | | | 2,718 | | | 2,303 | | | 1,257 | | | 0,811 | | | 2,261 | | | 0,973 | | | 1,942 | | | 1,821 | | | 1,269 | | 0,527 |
| -1 | | | 2,654 | | | 1,126 | | | 2,712 | | | 2,298 | | | 1,254 | | | 0,810 | | | 2,265 | | | 0,968 | | | 1,938 | | | 1,821 | | | 1,270 | | 0,531 |
| 0 | | | 2,648 | | | 1,124 | | | 2,707 | | | 2,294 | | | 1,252 | | | 0,808 | | | 2,268 | | | 0,962 | | | 1,934 | | | 1,821 | | | 1,271 | | 0,534 |
| 1 | | | 2,644 | | | 1,122 | | | 2,702 | | | 2,290 | | | 1,250 | | | 0,807 | | | 2,272 | | | 0,956 | | | 1,931 | | | 1,821 | | | 1,271 | | 0,538 |
| 2 | | | 2,640 | | | 1,120 | | | 2,698 | | | 2,286 | | | 1,248 | | | 0,805 | | | 2,276 | | | 0,950 | | | 1,928 | | | 1,820 | | | 1,272 | | 0,541 |
| 3 | | | 2,636 | | | 1,119 | | | 2,694 | | | 2,283 | | | 1,246 | | | 0,804 | | | 2,279 | | | 0,943 | | | 1,925 | | | 1,820 | | | 1,272 | | 0,544 |
| 4 | | | 2,633 | | | 1,117 | | | 2,690 | | | 2,280 | | | 1,244 | | | 0,803 | | | 2,283 | | | 0,936 | | | 1,922 | | | 1,819 | | | 1,272 | | 0,548 |
| 5 | | | 2,630 | | | 1,116 | | | 2,688 | | | 2,277 | | | 1,243 | | | 0,802 | | | 2,286 | | | 0,929 | | | 1,920 | | | 1,818 | | | 1,272 | | 0,551 |
