# Supplementary material for: A Biomechanical Analysis of Muscle Force Changes After Bilateral Sagittal Split Osteotomy
Source: Front Physiol. 2021 Jun 3;12:679644. doi: 10.3389/fphys.2021.679644 (PMC8209381; doi:10.3389/fphys.2021.679644)
Supplement: Supplementary file 4 [file Table_4.docx]

Supplementary Table 4. Calculations for assemblies of simple cases and those obtained as a result of numerical simulation

|  | | | SM_w_ | | DM_w_ | | MP_w_ | | AT_w_ | | MT_w_ | | PT_w_ | | SM_b_ | | DM_b_ | | MP_b_ | | AT_b_ | | MT_b_ | | PT_b_ | |
| --- | --- | --- | --- | --- | --- | --- | --- | --- | --- | --- | --- | --- | --- | --- | --- | --- | --- | --- | --- | --- | --- | --- | --- | --- | --- | --- |
| Assembly1 |  | 3,045 | | 1,283 | | 2,819 | | 2,606 | | 1,414 | | 0,932 | | 2,581 | | 1,117 | | 2,015 | | 2,085 | | 1,456 | | 0,611 | |  |
| DTr | 6 | 3,046 | | 1,293 | | 2,863 | | 2,638 | | 1,440 | | 0,929 | | 2,609 | | 1,106 | | 2,047 | | 2,094 | | 1,461 | | 0,614 | |  |
| Roll_w_ | 3 | 2,657 | | 1,146 | | 2,701 | | 2,286 | | 1,240 | | 0,803 | | 2,264 | | 0,960 | | 1,930 | | 1,817 | | 1,268 | | 0,533 | |  |
| Pitch_w_ | 3 | 2,658 | | 1,101 | | 2,691 | | 2,290 | | 1,252 | | 0,822 | | 2,255 | | 0,957 | | 1,923 | | 1,810 | | 1,263 | | 0,531 | |  |
| Yaw_b_ | 3 | 2,631 | | 1,117 | | 2,689 | | 2,278 | | 1,244 | | 0,803 | | 2,261 | | 0,974 | | 1,921 | | 1,830 | | 1,280 | | 0,537 | |  |
| Roll_b_ | 1 | 2,647 | | 1,123 | | 2,705 | | 2,292 | | 1,251 | | 0,808 | | 2,272 | | 0,968 | | 1,933 | | 1,819 | | 1,267 | | 0,533 | |  |
| S0 |  | 2,648 | | 1,124 | | 2,707 | | 2,294 | | 1,252 | | 0,808 | | 2,268 | | 0,962 | | 1,934 | | 1,821 | | 1,271 | | 0,534 | |  |
| ∑Diff |  | 0,397 | | 0,159 | | 0,116 | | 0,317 | | 0,167 | | 0,124 | | 0,318 | | 0,155 | | 0,084 | | 0,266 | | 0,186 | | 0,397 | |  |
| S0+∑Diff |  | 3,045 | | 1,283 | | 2,823 | | 2,610 | | 1,419 | | 0,932 | | 2,587 | | 1,117 | | 2,018 | | 2,087 | | 1,457 | | 3,045 | |  |
| % |  | 0,019 | | 0,060 | | 0,142 | | 0,180 | | 0,292 | | 0,027 | | 0,232 | | -0,017 | | 0,138 | | 0,100 | | 0,067 | | 0,019 | |  |
|  | | | SM_w_ | | DM_w_ | | MP_w_ | | AT_w_ | | MT_w_ | | PT_w_ | | SM_b_ | | DM_b_ | | MP_b_ | | AT_b_ | | MT_b_ | | PT_b_ | |
| Assembly2 |  | 3,020 | | 1,266 | | 2,805 | | 2,559 | | 1,383 | | 0,913 | | 2,563 | | 1,105 | | 2,005 | | 2,022 | | 1,393 | | 0,589 | |  |
| DRot | -6 | 2,580 | | 1,095 | | 2,643 | | 2,235 | | 1,220 | | 0,787 | | 2,210 | | 0,937 | | 1,888 | | 1,774 | | 1,238 | | 0,520 | |  |
| DTr | 6 | 3,046 | | 1,293 | | 2,863 | | 2,638 | | 1,440 | | 0,929 | | 2,609 | | 1,106 | | 2,047 | | 2,094 | | 1,461 | | 0,614 | |  |
| Yaw_w_ | -2 | 2,657 | | 1,116 | | 2,723 | | 2,286 | | 1,246 | | 0,806 | | 2,282 | | 0,968 | | 1,946 | | 1,832 | | 1,278 | | 0,537 | |  |
| Roll_w_ | -4 | 2,659 | | 1,153 | | 2,700 | | 2,285 | | 1,236 | | 0,801 | | 2,263 | | 0,960 | | 1,929 | | 1,817 | | 1,268 | | 0,533 | |  |
| Pitch_w_ | 3 | 2,658 | | 1,101 | | 2,691 | | 2,290 | | 1,252 | | 0,822 | | 2,255 | | 0,957 | | 1,923 | | 1,810 | | 1,263 | | 0,531 | |  |
| Yaw_b_ | -2 | 2,662 | | 1,130 | | 2,721 | | 2,305 | | 1,258 | | 0,812 | | 2,274 | | 0,954 | | 1,944 | | 1,813 | | 1,264 | | 0,532 | |  |
| Roll_b_ | 5 | 2,643 | | 1,122 | | 2,701 | | 2,289 | | 1,249 | | 0,806 | | 2,280 | | 0,993 | | 1,930 | | 1,814 | | 1,252 | | 0,529 | |  |
| S0 |  | 2,648 | | 1,124 | | 2,707 | | 2,294 | | 1,252 | | 0,808 | | 2,268 | | 0,962 | | 1,934 | | 1,821 | | 1,271 | | 0,534 | |  |
| ∑Diff |  | 0,367 | | 0,141 | | 0,096 | | 0,272 | | 0,138 | | 0,108 | | 0,294 | | 0,141 | | 0,069 | | 0,208 | | 0,129 | | 0,058 | |  |
| S0+∑Diff |  | 3,015 | | 1,265 | | 2,803 | | 2,565 | | 1,390 | | 0,916 | | 2,562 | | 1,103 | | 2,003 | | 2,029 | | 1,400 | | 0,592 | |  |
| % |  | -0,175 | | -0,068 | | -0,099 | | 0,225 | | 0,512 | | 0,253 | | -0,043 | | -0,224 | | -0,102 | | 0,301 | | 0,553 | | 0,528 | |  |
|  | | | SM_w_ | | DM_w_ | | MP_w_ | | AT_w_ | | MT_w_ | | PT_w_ | | SM_b_ | | DM_b_ | | MP_b_ | | AT_b_ | | MT_b_ | | PT_b_ | |
| Assembly3 |  | 2,876 | | 1,233 | | 2,786 | | 2,479 | | 1,347 | | 0,871 | | 2,475 | | 1,033 | | 1,991 | | 1,955 | | 1,357 | | 0,579 | |  |
| DRot | -3 | 2,609 | | 1,107 | | 2,670 | | 2,259 | | 1,233 | | 0,796 | | 2,235 | | 0,948 | | 1,908 | | 1,794 | | 1,252 | | 0,526 | |  |
| DTr | 4 | 2,908 | | 1,235 | | 2,823 | | 2,518 | | 1,375 | | 0,887 | | 2,491 | | 1,056 | | 2,018 | | 1,999 | | 1,395 | | 0,586 | |  |
| Roll_w_ | 2 | 2,655 | | 1,138 | | 2,703 | | 2,288 | | 1,244 | | 0,804 | | 2,265 | | 0,961 | | 1,931 | | 1,818 | | 1,269 | | 0,533 | |  |
| Yaw_b_ | -2 | 2,662 | | 1,130 | | 2,721 | | 2,305 | | 1,258 | | 0,812 | | 2,274 | | 0,954 | | 1,944 | | 1,813 | | 1,264 | | 0,532 | |  |
| Roll_b_ | 2 | 2,645 | | 1,123 | | 2,703 | | 2,291 | | 1,250 | | 0,807 | | 2,275 | | 0,975 | | 1,932 | | 1,817 | | 1,263 | | 0,532 | |  |
| Pitch_b_ | 2 | 2,640 | | 1,120 | | 2,698 | | 2,286 | | 1,248 | | 0,805 | | 2,276 | | 0,950 | | 1,928 | | 1,820 | | 1,272 | | 0,541 | |  |
| S0 |  | 2,648 | | 1,124 | | 2,707 | | 2,294 | | 1,252 | | 0,808 | | 2,268 | | 0,962 | | 1,934 | | 1,821 | | 1,271 | | 0,534 | |  |
| ∑Diff |  | 0,228 | | 0,108 | | 0,078 | | 0,187 | | 0,097 | | 0,064 | | 0,204 | | 0,071 | | 0,056 | | 0,136 | | 0,090 | | 0,046 | |  |
| S0+∑Diff |  | 2,876 | | 1,233 | | 2,784 | | 2,480 | | 1,348 | | 0,872 | | 2,472 | | 1,033 | | 1,990 | | 1,957 | | 1,360 | | 0,580 | |  |
| % |  | 0,022 | | -0,055 | | -0,050 | | 0,058 | | 0,091 | | 0,076 | | -0,093 | | 0,034 | | -0,048 | | 0,115 | | 0,237 | | 0,123 | |  |
|  | | | SM_w_ | | DM_w_ | | MP_w_ | | AT_w_ | | MT_w_ | | PT_w_ | | SM_b_ | | DM_b_ | | MP_b_ | | AT_b_ | | MT_b_ | | PT_b_ | |
| Assembly4 |  | 2,396 | | 1,040 | | 2,572 | | 2,107 | | 1,154 | | 0,730 | | 2,080 | | 0,877 | | 1,837 | | 1,653 | | 1,150 | | 0,484 | |  |
| DTr | -4 | 2,408 | | 1,022 | | 2,551 | | 2,085 | | 1,138 | | 0,735 | | 2,063 | | 0,875 | | 1,822 | | 1,656 | | 1,155 | | 0,486 | |  |
| Yaw_w_ | 2 | 2,640 | | 1,132 | | 2,692 | | 2,300 | | 1,257 | | 0,810 | | 2,256 | | 0,957 | | 1,924 | | 1,811 | | 1,264 | | 0,531 | |  |
| Roll_w_ | -2 | 2,640 | | 1,110 | | 2,713 | | 2,300 | | 1,261 | | 0,812 | | 2,273 | | 0,964 | | 1,938 | | 1,825 | | 1,274 | | 0,535 | |  |
| Pitch_w_ | -3 | 2,639 | | 1,145 | | 2,727 | | 2,296 | | 1,251 | | 0,794 | | 2,285 | | 0,969 | | 1,948 | | 1,834 | | 1,280 | | 0,538 | |  |
| Yaw_b_ | -2 | 2,662 | | 1,130 | | 2,721 | | 2,305 | | 1,258 | | 0,812 | | 2,274 | | 0,954 | | 1,944 | | 1,813 | | 1,264 | | 0,532 | |  |
| Roll_b_ | 1 | 2,647 | | 1,123 | | 2,705 | | 2,292 | | 1,251 | | 0,808 | | 2,272 | | 0,968 | | 1,933 | | 1,819 | | 1,267 | | 0,533 | |  |
| S0 |  | 2,648 | | 1,124 | | 2,707 | | 2,294 | | 1,252 | | 0,808 | | 2,268 | | 0,962 | | 1,934 | | 1,821 | | 1,271 | | 0,534 | |  |
| ∑Diff |  | -0,255 | | -0,083 | | -0,132 | | -0,183 | | -0,095 | | -0,078 | | -0,187 | | -0,085 | | -0,095 | | -0,167 | | -0,121 | | -0,049 | |  |
| S0+∑Diff |  | 2,393 | | 1,041 | | 2,574 | | 2,110 | | 1,157 | | 0,731 | | 2,081 | | 0,878 | | 1,839 | | 1,654 | | 1,150 | | 0,485 | |  |
| % |  | -0,122 | | 0,115 | | 0,082 | | 0,150 | | 0,295 | | 0,098 | | 0,078 | | 0,027 | | 0,080 | | 0,025 | | 0,017 | | 0,048 | |  |

Supplementary 4. continued

|  | | | SM_w_ | | | | DM_w_ | | | | MP_w_ | | | | AT_w_ | | | | MT_w_ | | | | PT_w_ | | | | SM_b_ | | | | DM_b_ | | | | MP_b_ | | | | AT_b_ | | | | MT_b_ | | | | PT_b_ | | | |  |  |
| --- | --- | --- | --- | --- | --- | --- | --- | --- | --- | --- | --- | --- | --- | --- | --- | --- | --- | --- | --- | --- | --- | --- | --- | --- | --- | --- | --- | --- | --- | --- | --- | --- | --- | --- | --- | --- | --- | --- | --- | --- | --- | --- | --- | --- | --- | --- | --- | --- | --- | --- | --- | --- |
| Assembly5 |  | 3,181 | | | | 1,321 | | | | 2,838 | | | | 2,706 | | | | 1,468 | | | | 0,974 | | | | 2,694 | | | | 1,168 | | | | 2,029 | | | | 2,135 | | | | 1,472 | | | | 0,622 | | | |  |  |  |
| DRot | -3 | 2,609 | | | | 1,107 | | | | 2,670 | | | | 2,259 | | | | 1,233 | | | | 0,796 | | | | 2,235 | | | | 0,948 | | | | 1,908 | | | | 1,794 | | | | 1,252 | | | | 0,526 | | | |  |  |  |
| DTr | 8 | 3,189 | | | | 1,354 | | | | 2,889 | | | | 2,761 | | | | 1,507 | | | | 0,973 | | | | 2,731 | | | | 1,158 | | | | 2,066 | | | | 2,192 | | | | 1,530 | | | | 0,643 | | | |  |  |  |
| Yaw_w_ | -1 | 2,653 | | | | 1,120 | | | | 2,715 | | | | 2,290 | | | | 1,249 | | | | 0,807 | | | | 2,275 | | | | 0,965 | | | | 1,940 | | | | 1,826 | | | | 1,274 | | | | 0,536 | | | |  |  |  |
| Roll_w_ | 3 | 2,657 | | | | 1,146 | | | | 2,701 | | | | 2,286 | | | | 1,240 | | | | 0,803 | | | | 2,264 | | | | 0,960 | | | | 1,930 | | | | 1,817 | | | | 1,268 | | | | 0,533 | | | |  |  |  |
| Pitch_w_ | 4 | 2,662 | | | | 1,093 | | | | 2,687 | | | | 2,288 | | | | 1,252 | | | | 0,827 | | | | 2,252 | | | | 0,955 | | | | 1,920 | | | | 1,808 | | | | 1,262 | | | | 0,530 | | | |  |  |  |
| Yaw_b_ | 1 | 2,655 | | | | 1,127 | | | | 2,713 | | | | 2,299 | | | | 1,255 | | | | 0,810 | | | | 2,271 | | | | 0,958 | | | | 1,939 | | | | 1,817 | | | | 1,267 | | | | 0,533 | | | |  |  |  |
| Roll_b_ | 5 | 2,643 | | | | 1,122 | | | | 2,701 | | | | 2,289 | | | | 1,249 | | | | 0,806 | | | | 2,280 | | | | 0,993 | | | | 1,930 | | | | 1,814 | | | | 1,252 | | | | 0,529 | | | |  |  |  |
| S0 |  | 2,648 | | | | 1,124 | | | | 2,707 | | | | 2,294 | | | | 1,252 | | | | 0,808 | | | | 2,268 | | | | 0,962 | | | | 1,934 | | | | 1,821 | | | | 1,271 | | | | 0,534 | | | |  |  |  |
| ∑Diff |  | 0,528 | | | | 0,199 | | | | 0,130 | | | | 0,418 | | | | 0,222 | | | | 0,165 | | | | 0,428 | | | | 0,203 | | | | 0,094 | | | | 0,322 | | | | 0,210 | | | | 0,091 | | | |  |  |  |
| S0+∑Diff |  | 3,177 | | | | 1,324 | | | | 2,837 | | | | 2,712 | | | | 1,474 | | | | 0,973 | | | | 2,696 | | | | 1,165 | | | | 2,028 | | | | 2,143 | | | | 1,481 | | | | 0,625 | | | |  |  |  |
| % |  | -0,125 | | | | 0,181 | | | | -0,053 | | | | 0,230 | | | | 0,437 | | | | -0,011 | | | | 0,083 | | | | -0,246 | | | | -0,059 | | | | 0,354 | | | | 0,598 | | | | 0,533 | | | |  |  |  |
|  | | | | | SM_w_ | | | | DM_w_ | | | | MP_w_ | | | | AT_w_ | | | | MT_w_ | | | | PT_w_ | | | | SM_b_ | | | | DM_b_ | | | | MP_b_ | | | | AT_b_ | | | | MT_b_ | | | | PT_b_ | | | |
| Assembly6 |  | | | 2,406 | | | | 1,050 | | | | 2,573 | | | | 2,120 | | | | 1,161 | | | | 0,734 | | | | 2,111 | | | | 0,883 | | | | 1,838 | | | | 1,696 | | | | 1,187 | | | | 0,507 | | | |  |
| DRot | 4 | | | 2,695 | | | | 1,144 | | | | 2,747 | | | | 2,334 | | | | 1,274 | | | | 0,822 | | | | 2,308 | | | | 0,979 | | | | 1,963 | | | | 1,853 | | | | 1,293 | | | | 0,544 | | | |  |
| DTr | -4 | | | 2,408 | | | | 1,022 | | | | 2,551 | | | | 2,085 | | | | 1,138 | | | | 0,735 | | | | 2,063 | | | | 0,875 | | | | 1,822 | | | | 1,656 | | | | 1,155 | | | | 0,486 | | | |  |
| Yaw_w_ | 3 | | | 2,637 | | | | 1,137 | | | | 2,686 | | | | 2,302 | | | | 1,260 | | | | 0,812 | | | | 2,251 | | | | 0,955 | | | | 1,919 | | | | 1,807 | | | | 1,261 | | | | 0,530 | | | |  |
| Roll_w_ | -2 | | | 2,640 | | | | 1,110 | | | | 2,713 | | | | 2,300 | | | | 1,261 | | | | 0,812 | | | | 2,273 | | | | 0,964 | | | | 1,938 | | | | 1,825 | | | | 1,274 | | | | 0,535 | | | |  |
| Pitch_w_ | -3 | | | 2,639 | | | | 1,145 | | | | 2,727 | | | | 2,296 | | | | 1,251 | | | | 0,794 | | | | 2,285 | | | | 0,969 | | | | 1,948 | | | | 1,834 | | | | 1,280 | | | | 0,538 | | | |  |
| Yaw_b_ | 2 | | | 2,637 | | | | 1,119 | | | | 2,694 | | | | 2,283 | | | | 1,246 | | | | 0,804 | | | | 2,263 | | | | 0,970 | | | | 1,925 | | | | 1,827 | | | | 1,277 | | | | 0,536 | | | |  |
| Pitch_b_ | 3 | | | 2,636 | | | | 1,119 | | | | 2,694 | | | | 2,283 | | | | 1,246 | | | | 0,804 | | | | 2,279 | | | | 0,943 | | | | 1,925 | | | | 1,820 | | | | 1,272 | | | | 0,544 | | | |  |
| S0 |  | | | 2,648 | | | | 1,124 | | | | 2,707 | | | | 2,294 | | | | 1,252 | | | | 0,808 | | | | 2,268 | | | | 0,962 | | | | 1,934 | | | | 1,821 | | | | 1,271 | | | | 0,534 | | | |  |
| ∑Diff |  | | | -0,248 | | | | -0,074 | | | | -0,136 | | | | -0,172 | | | | -0,087 | | | | -0,073 | | | | -0,156 | | | | -0,080 | | | | -0,097 | | | | -0,124 | | | | -0,083 | | | | -0,026 | | | |  |
| S0+∑Diff |  | | | 2,400 | | | | 1,050 | | | | 2,571 | | | | 2,122 | | | | 1,164 | | | | 0,735 | | | | 2,113 | | | | 0,883 | | | | 1,837 | | | | 1,696 | | | | 1,188 | | | | 0,508 | | | |  |
| % |  | | | -0,262 | | | | 0,038 | | | | -0,068 | | | | 0,082 | | | | 0,258 | | | | 0,066 | | | | 0,074 | | | | -0,092 | | | | -0,070 | | | | 0,051 | | | | 0,052 | | | | 0,192 | | | |  |
|  | | | | | SM_w_ | | | | DM_w_ | | | | MP_w_ | | | | AT_w_ | | | | MT_w_ | | | | PT_w_ | | | | SM_b_ | | | | DM_b_ | | | | MP_b_ | | | | AT_b_ | | | | MT_b_ | | | | PT_b_ | | | |
| Assembly7 |  | | | 2,266 | | | | 0,948 | | | | 2,456 | | | | 1,982 | | | | 1,088 | | | | 0,701 | | | | 1,922 | | | | 0,853 | | | | 1,754 | | | | 1,575 | | | | 1,103 | | | | 0,449 | | | |  |
| DRot | -2 | | | 2,622 | | | | 1,113 | | | | 2,683 | | | | 2,271 | | | | 1,240 | | | | 0,800 | | | | 2,246 | | | | 0,953 | | | | 1,917 | | | | 1,803 | | | | 1,258 | | | | 0,529 | | | |  |
| DTr | -6 | | | 2,294 | | | | 0,973 | | | | 2,461 | | | | 1,987 | | | | 1,084 | | | | 0,700 | | | | 1,965 | | | | 0,833 | | | | 1,758 | | | | 1,577 | | | | 1,101 | | | | 0,463 | | | |  |
| Roll_w_ | -3 | | | 2,634 | | | | 1,103 | | | | 2,716 | | | | 2,304 | | | | 1,265 | | | | 0,814 | | | | 2,276 | | | | 0,965 | | | | 1,941 | | | | 1,827 | | | | 1,275 | | | | 0,536 | | | |  |
| Yaw_b_ | 4 | | | 2,626 | | | | 1,115 | | | | 2,684 | | | | 2,274 | | | | 1,241 | | | | 0,801 | | | | 2,258 | | | | 0,978 | | | | 1,918 | | | | 1,832 | | | | 1,283 | | | | 0,538 | | | |  |
| Roll_b_ | -2 | | | 2,653 | | | | 1,126 | | | | 2,712 | | | | 2,298 | | | | 1,254 | | | | 0,810 | | | | 2,260 | | | | 0,949 | | | | 1,937 | | | | 1,825 | | | | 1,279 | | | | 0,537 | | | |  |
| Pitch_b_ | -5 | | | 2,678 | | | | 1,137 | | | | 2,737 | | | | 2,319 | | | | 1,266 | | | | 0,817 | | | | 2,250 | | | | 0,988 | | | | 1,955 | | | | 1,819 | | | | 1,266 | | | | 0,517 | | | |  |
| S0 |  | | | 2,648 | | | | 1,124 | | | | 2,707 | | | | 2,294 | | | | 1,252 | | | | 0,808 | | | | 2,268 | | | | 0,962 | | | | 1,934 | | | | 1,821 | | | | 1,271 | | | | 0,534 | | | |  |
| ∑Diff |  | | | -0,383 | | | | -0,178 | | | | -0,248 | | | | -0,309 | | | | -0,161 | | | | -0,106 | | | | -0,354 | | | | -0,105 | | | | -0,178 | | | | -0,242 | | | | -0,162 | | | | -0,085 | | | |  |
| S0+∑Diff |  | | | 2,265 | | | | 0,946 | | | | 2,459 | | | | 1,984 | | | | 1,091 | | | | 0,702 | | | | 1,914 | | | | 0,857 | | | | 1,756 | | | | 1,579 | | | | 1,109 | | | | 0,449 | | | |  |
| % |  | | | -0,031 | | | | -0,258 | | | | 0,114 | | | | 0,128 | | | | 0,224 | | | | 0,181 | | | | -0,402 | | | | 0,480 | | | | 0,122 | | | | 0,253 | | | | 0,542 | | | | -0,015 | | | |  |
|  | | | | | SM_w_ | | | | DM_w_ | | | | MP_w_ | | | | AT_w_ | | | | MT_w_ | | | | PT_w_ | | | | SM_b_ | | | | DM_b_ | | | | MP_b_ | | | | AT_b_ | | | | MT_b_ | | | | PT_b_ | | | |
| Assembly8 |  | | | 2,153 | | | | 0,941 | | | | 2,345 | | | | 1,885 | | | | 1,031 | | | | 0,657 | | | | 1,854 | | | | 0,740 | | | | 1,675 | | | | 1,472 | | | | 1,032 | | | | 0,445 | | | |  |
| DRot | -2 | | | 2,622 | | | | 1,113 | | | | 2,683 | | | | 2,271 | | | | 1,240 | | | | 0,800 | | | | 2,246 | | | | 0,953 | | | | 1,917 | | | | 1,803 | | | | 1,258 | | | | 0,529 | | | |  |
| DTr | -8 | | | 2,183 | | | | 0,926 | | | | 2,366 | | | | 1,891 | | | | 1,032 | | | | 0,666 | | | | 1,870 | | | | 0,793 | | | | 1,689 | | | | 1,501 | | | | 1,048 | | | | 0,441 | | | |  |
| Yaw_w_ | 3 | | | 2,637 | | | | 1,137 | | | | 2,686 | | | | 2,302 | | | | 1,260 | | | | 0,812 | | | | 2,251 | | | | 0,955 | | | | 1,919 | | | | 1,807 | | | | 1,261 | | | | 0,530 | | | |  |
| Pitch_w_ | -2 | | | 2,642 | | | | 1,138 | | | | 2,720 | | | | 2,295 | | | | 1,251 | | | | 0,799 | | | | 2,279 | | | | 0,967 | | | | 1,943 | | | | 1,830 | | | | 1,277 | | | | 0,537 | | | |  |
| Yaw_b_ | -3 | | | 2,669 | | | | 1,133 | | | | 2,728 | | | | 2,311 | | | | 1,262 | | | | 0,814 | | | | 2,277 | | | | 0,951 | | | | 1,949 | | | | 1,808 | | | | 1,260 | | | | 0,531 | | | |  |
| Roll_b_ | 2 | | | 2,653 | | | | 1,126 | | | | 2,712 | | | | 2,298 | | | | 1,254 | | | | 0,810 | | | | 2,260 | | | | 0,949 | | | | 1,937 | | | | 1,825 | | | | 1,279 | | | | 0,537 | | | |  |
| Pitch_b_ | 4 | | | 2,633 | | | | 1,117 | | | | 2,690 | | | | 2,280 | | | | 1,244 | | | | 0,803 | | | | 2,283 | | | | 0,936 | | | | 1,922 | | | | 1,819 | | | | 1,272 | | | | 0,548 | | | |  |
| S0 |  | | | 2,648 | | | | 1,124 | | | | 2,707 | | | | 2,294 | | | | 1,252 | | | | 0,808 | | | | 2,268 | | | | 0,962 | | | | 1,934 | | | | 1,821 | | | | 1,271 | | | | 0,534 | | | |  |
| ∑Diff |  | | | -0,500 | | | | -0,178 | | | | -0,363 | | | | -0,407 | | | | -0,220 | | | | -0,153 | | | | -0,412 | | | | -0,231 | | | | -0,261 | | | | -0,353 | | | | -0,240 | | | | -0,088 | | | |  |
| S0+∑Diff |  | | | 2,148 | | | | 0,946 | | | | 2,344 | | | | 1,887 | | | | 1,032 | | | | 0,655 | | | | 1,856 | | | | 0,731 | | | | 1,673 | | | | 1,468 | | | | 1,030 | | | | 0,446 | | | |  |
| % |  | | | -0,208 | | | | 0,517 | | | | -0,058 | | | | 0,080 | | | | 0,126 | | | | -0,190 | | | | 0,126 | | | | -1,122 | | | | -0,074 | | | | -0,237 | | | | -0,191 | | | | 0,302 | | | |  |

Supplementary Table 4. continued 2

|  | | | | SM_w_ | | | DM_w_ | | | MP_w_ | | | AT_w_ | | | MT_w_ | | | PT_w_ | | | SM_b_ | | | DM_b_ | | | MP_b_ | | | AT_b_ | | | MT_b_ | | | PT_b_ | | |
| --- | --- | --- | --- | --- | --- | --- | --- | --- | --- | --- | --- | --- | --- | --- | --- | --- | --- | --- | --- | --- | --- | --- | --- | --- | --- | --- | --- | --- | --- | --- | --- | --- | --- | --- | --- | --- | --- | --- | --- |
| Assembly9 |  | | 2,777 | | | 1,156 | | | 2,716 | | | 2,363 | | | 1,281 | | | 0,855 | | | 2,309 | | | 0,972 | | | 1,941 | | | 1,837 | | | 1,279 | | | 0,536 | | |  |
| DRot | -4 | | 2,595 | | | 1,101 | | | 2,657 | | | 2,247 | | | 1,227 | | | 0,792 | | | 2,223 | | | 0,943 | | | 1,898 | | | 1,784 | | | 1,245 | | | 0,523 | | |  |
| DTr | 2 | | 2,776 | | | 1,178 | | | 2,771 | | | 2,404 | | | 1,312 | | | 0,847 | | | 2,377 | | | 1,008 | | | 1,980 | | | 1,908 | | | 1,332 | | | 0,560 | | |  |
| Roll_w_ | 4 | | 2,659 | | | 1,153 | | | 2,700 | | | 2,285 | | | 1,236 | | | 0,801 | | | 2,263 | | | 0,960 | | | 1,929 | | | 1,817 | | | 1,268 | | | 0,533 | | |  |
| Pitch_w_ | 5 | | 2,665 | | | 1,084 | | | 2,684 | | | 2,286 | | | 1,251 | | | 0,832 | | | 2,249 | | | 0,954 | | | 1,917 | | | 1,805 | | | 1,260 | | | 0,530 | | |  |
| Yaw_b_ | -3 | | 2,669 | | | 1,133 | | | 2,728 | | | 2,311 | | | 1,262 | | | 0,814 | | | 2,277 | | | 0,951 | | | 1,949 | | | 1,808 | | | 1,260 | | | 0,531 | | |  |
| Pitch_b_ | -1 | | 2,654 | | | 1,126 | | | 2,712 | | | 2,298 | | | 1,254 | | | 0,810 | | | 2,265 | | | 0,968 | | | 1,938 | | | 1,821 | | | 1,270 | | | 0,531 | | |  |
| S0 |  | | 2,648 | | | 1,124 | | | 2,707 | | | 2,294 | | | 1,252 | | | 0,808 | | | 2,268 | | | 0,962 | | | 1,934 | | | 1,821 | | | 1,271 | | | 0,534 | | |  |
| ∑Diff |  | | 0,127 | | | 0,031 | | | 0,011 | | | 0,070 | | | 0,031 | | | 0,047 | | | 0,043 | | | 0,011 | | | 0,008 | | | 0,018 | | | 0,010 | | | 0,002 | | |  |
| S0+∑Diff |  | | 2,775 | | | 1,155 | | | 2,718 | | | 2,364 | | | 1,283 | | | 0,855 | | | 2,312 | | | 0,973 | | | 1,942 | | | 1,839 | | | 1,281 | | | 0,536 | | |  |
| % |  | | -0,052 | | | -0,131 | | | 0,062 | | | 0,023 | | | 0,164 | | | 0,027 | | | 0,131 | | | 0,118 | | | 0,063 | | | 0,125 | | | 0,123 | | | 0,137 | | |  |
|  | | | | SM_w_ | | | DM_w_ | | | MP_w_ | | | AT_w_ | | | MT_w_ | | | PT_w_ | | | SM_b_ | | | DM_b_ | | | MP_b_ | | | AT_b_ | | | MT_b_ | | | PT_b_ | | |
| Assembly10 |  | 3,305 | | | 1,396 | | | 2,826 | | | 2,856 | | | 1,560 | | | 1,031 | | | 2,808 | | | 1,179 | | | 2,020 | | | 2,200 | | | 1,518 | | | 0,649 | | |  |  |
| DRot | -3 | 2,609 | | | 1,107 | | | 2,670 | | | 2,259 | | | 1,233 | | | 0,796 | | | 2,235 | | | 0,948 | | | 1,908 | | | 1,794 | | | 1,252 | | | 0,526 | | |  |  |
| DTr | 10 | 3,337 | | | 1,417 | | | 2,900 | | | 2,890 | | | 1,577 | | | 1,018 | | | 2,858 | | | 1,212 | | | 2,074 | | | 2,294 | | | 1,601 | | | 0,673 | | |  |  |
| Yaw_w_ | 3 | 2,637 | | | 1,137 | | | 2,686 | | | 2,302 | | | 1,260 | | | 0,812 | | | 2,251 | | | 0,955 | | | 1,919 | | | 1,807 | | | 1,261 | | | 0,530 | | |  |  |
| Roll_w_ | 2 | 2,655 | | | 1,138 | | | 2,703 | | | 2,288 | | | 1,244 | | | 0,804 | | | 2,265 | | | 0,961 | | | 1,931 | | | 1,818 | | | 1,269 | | | 0,533 | | |  |  |
| Pitch_w_ | 4 | 2,662 | | | 1,093 | | | 2,687 | | | 2,288 | | | 1,252 | | | 0,827 | | | 2,252 | | | 0,955 | | | 1,920 | | | 1,808 | | | 1,262 | | | 0,530 | | |  |  |
| Yaw_b_ | -3 | 2,669 | | | 1,133 | | | 2,728 | | | 2,311 | | | 1,262 | | | 0,814 | | | 2,277 | | | 0,951 | | | 1,949 | | | 1,808 | | | 1,260 | | | 0,531 | | |  |  |
| Roll_b_ | 4 | 2,643 | | | 1,122 | | | 2,702 | | | 2,289 | | | 1,249 | | | 0,807 | | | 2,278 | | | 0,987 | | | 1,930 | | | 1,815 | | | 1,256 | | | 0,530 | | |  |  |
| Pitch_b_ | 2 | 2,640 | | | 1,120 | | | 2,698 | | | 2,286 | | | 1,248 | | | 0,805 | | | 2,276 | | | 0,950 | | | 1,928 | | | 1,820 | | | 1,272 | | | 0,541 | | |  |  |
| S0 |  | 2,648 | | | 1,124 | | | 2,707 | | | 2,294 | | | 1,252 | | | 0,808 | | | 2,268 | | | 0,962 | | | 1,934 | | | 1,821 | | | 1,271 | | | 0,534 | | |  |  |
| ∑Diff |  | 0,663 | | | 0,274 | | | 0,120 | | | 0,566 | | | 0,309 | | | 0,219 | | | 0,544 | | | 0,221 | | | 0,087 | | | 0,397 | | | 0,265 | | | 0,121 | | |  |  |
| S0+∑Diff |  | 3,312 | | | 1,398 | | | 2,826 | | | 2,859 | | | 1,561 | | | 1,027 | | | 2,812 | | | 1,183 | | | 2,020 | | | 2,218 | | | 1,536 | | | 0,655 | | |  |  |
| % |  | 0,210 | | | 0,173 | | | 0,011 | | | 0,135 | | | 0,052 | | | -0,401 | | | 0,156 | | | 0,326 | | | 0,023 | | | 0,801 | | | 1,199 | | | 0,884 | | |  |  |
